# Mobile Health Apps for Patient-Centered Care: Review of United States Rheumatoid Arthritis Apps for Engagement and Activation

**DOI:** 10.2196/39881

**Published:** 2022-12-05

**Authors:** Melanie J Cozad, Marissa Crum, Hannah Tyson, Perry R Fleming, Jeanine Stratton, Ann Blair Kennedy, Lisa C Lindley, Ronnie D Horner

**Affiliations:** 1 Health Services Research and Administration Department College of Public Health University of Nebraska Medical Center Omaha, NE United States; 2 School of Medicine Greenville University of South Carolina Greenville, SC United States; 3 Business and Accounting Department Furman University Greenville, SC United States; 4 School of Medicine Greenville University of South Carolina Columbia, SC United States; 5 College of Nursing University of Tennessee-Knoxville Knoxville, TN United States

**Keywords:** rheumatoid arthritis, rheumatism, arthritis, mobile app, mobile applications, mHealth, patient engagement, patient activation, patient-centered care, patient centred, person centered, Mobile Application Rating Scale, social cognitive theory, mobile health, review, systematic search, app feature, content analysis, functionality, user rating, patient rating

## Abstract

**Background:**

Rheumatoid arthritis (RA) is a highly dynamic and individualized disease in terms of its patterns of symptomatic flare-ups and periods of remission. Patient-centered care (PCC) aligns patients’ lifestyle goals with their preferences for managing symptoms and side effects through the selection of therapies appropriate for disease management. Mobile health (mHealth) apps have the potential to engage and activate patients in PCC. mHealth apps can provide features that increase disease knowledge, collect patient-generated health indicators and behavioral metrics, and highlight goals for disease management. However, little evidence-based guidance exists as to which apps contain functionality essential for supporting the delivery of PCC.

**Objective:**

The objective of this study was to evaluate the patient-centeredness of United States–based rheumatoid arthritis mobile apps in terms of patient engagement and activation.

**Methods:**

A search of mobile apps on 2 major United States app stores (Apple App Store and Google Play) was conducted from June 2020 to July 2021 to identify apps designed for use by patients with RA by adapting the PRISMA (Preferred Reporting Items for Systematic Reviews and Meta-analyses) guidelines for mobile health app screening based on the literature. Reviewers conducted a content analysis of mobile app features to evaluate their functionality for patient engagement and activation. Engagement and activation were assessed using the Mobile Application Rating Scale (MARS) and social cognitive theory, respectively. Apps were ranked by their ability to facilitate PCC care along 2 dimensions: engagement and activation.

**Results:**

A total of 202 mobile apps were initially identified, and 20 remained after screening. Two apps emerged with the greatest ability to facilitate PCC. Both apps were scored as having acceptable or good patient engagement according to the MARS. These 2 apps also had high patient activation according to social cognitive theory, with many features within those apps representing theoretical constructs such as knowledge, perceived self-efficacy, and expectations about outcomes that support behavioral management of RA.

**Conclusions:**

We found very few mobile apps available within the United States that have functionality that both engages and activates the patient to facilitate PCC. As the prevalence of mobile apps expands, the design of mobile apps needs to integrate patients to ensure that their functionality promotes engagement and activation. More research is needed to understand how mobile app use impacts patient engagement and activation, and ultimately, treatment decisions and disease trajectory.

## Introduction

Mobile health (mHealth) apps are emerging as an important approach to support the delivery of patient-centered care (PCC) for chronic conditions such as rheumatoid arthritis (RA). PCC seeks to integrate patient values into clinical decisions by encouraging active collaboration and shared therapeutic decisions between the patient and rheumatological provider [1]. This collaboration is imperative to managing the symptoms of RA more effectively, including chronic pain, fatigue, and joint inflammation, which affect 2.1 million people in the United States [2-4]. One-third of patients with RA experience alternating periods of disease control and relapse, and women are 2 to 3 more times likely to be afflicted than men [5,6].

To assist patients, numerous mHealth apps have been developed to increase knowledge of the disease, track problematic symptoms and side effects, and support social interactions [7,8]. Emerging evidence suggests that patients with RA are willing to adopt these apps [8]. Yet, despite their promise, there remains a lack of evidence guiding patients and health professionals as to which apps to adopt and use [9]. Several recent systematic reviews of RA apps in different countries focused on their ease of use and ability to support self-management of the disease [7,10-13]. The reviews uniformly reported a lack of high-quality apps that promote patient use and recommended more research to understand their efficacy [7,10-13]. Uncertainty also exists as to whether mHealth apps can improve patient-centered outcomes, including patient experience and satisfaction with care [9]. One challenge to assessing patient-centeredness is a lack of shared understanding about what constitutes relevant outcomes and how to evaluate them within the digital space [9,14,15].

mHealth apps that have the potential to advance PCC must demonstrate functionality to engage and activate the patient [9,14,16]. Engaged and activated patients collaborate with their rheumatologist, receive and internalize information related to their care, are involved in decision-making, and take the behavioral actions necessary to follow through on treatment plans [1,9]. These actions lead to improved patient experiences and satisfaction [17-19]. When applied to mobile app evaluation, the literature provides definitions of patient engagement and activation [9]. Patient engagement is the extent to which patients can use the app features (ie, amount, frequency, duration, and depth of usage) in addition to the user’s overall experience with the app [9]. Patient activation refers to the willingness and ability of patients to take behavioral actions to manage their RA and overall health [9]. In assessing mHealth apps, patients must perceive that the app has the features they desire to support them in taking behavioral actions to collaborate with providers and manage their RA between clinical visits [9]. With an increasing number of mHealth apps for RA available within the United States, patients and health care professionals need evidence-based guidance on which apps contain the functionality essential for improving the delivery of PCC. Therefore, the objective of this study was to evaluate the patient-centeredness of United States–based RA mHealth apps in terms of patient engagement and activation.

## Methods

### mHealth App Identification

To identify mobile apps that facilitate PCC, we conducted a systematic search of Apple (iOS) and Google Play (Android) stores in the United States by adopting the PRISMA (Preferred Reporting Items for Systematic Reviews and Meta-analyses) guidelines for a health app–focused review [20-23]. From June 1, 2020 to July 1, 2020, 2 independent reviewers (authors MC and HT) conducted searches of both stores using the terms “rheumatoid arthritis” OR “RA apps” OR “RA tracking” OR “RA management” OR “pain management” OR “pain tracking” OR “symptom tracking” OR “arthritis.” The app inclusion criteria were (1) smartphone apps that could run on iOS or Android software systems, (2) apps available for download in Apple and Google Play app stores within the United States, and (3) those specific to arthritis or RA and potentially relevant for use by a patient to manage their disease. After additional review, apps were excluded if they were (1) not intended for the target age group 18 years and up, (2) not in the English language, (3) a clinic tool intended for use only by providers, (4) provided only educational material from scientific journals and other resources, and (5) solely telehealth apps. Duplicates were examined based on the app logo and description. If the logo and description were identical, then 1 was removed. The 2 independent reviewers met to review the list of apps and discuss and reconcile any differences based on the inclusion and exclusion criteria. Reviewers came to a consensus on the final list of apps to download.

In July 2020, apps were downloaded in either the Android or iOS version depending on the device available to the reviewer. Android-only apps were downloaded and viewed using a Tracphone Alcatel TCL LX A502 smartphone. IOS apps were downloaded using iPhones (10 and 11) running software version iOS. Downloaded apps were further excluded after each of 2 independent reviewers verified that the app (1) was not recently updated and could not be opened and function, (2) had a feature or 2 that resulted in the app malfunctioning, (3) was removed from the Google Play and/or Apple stores during the study period, or (4) participation required a specific invitation from a research group. For the final set of apps, each reviewer completed the tutorial and navigated through the key features. The reviewers gathered operating characteristics for each app that included (1) app name, (2) logo, (3) operating system, (4) developer, (5) platform (ie, Apple or Android), (6) most recent version available or the date that the app was created, (7) price, (8) total number of features within the app, and (9) approximate number of downloads. Each independent reviewer reviewed the other’s work for consistency.

### Data Extraction

For the final set of apps, our team developed a data extraction process from August to December 2020 that evaluated patient engagement and activation of mHealth apps based on definitions and practices used within the mHealth literature [9,24]. Patient engagement is defined as the desire and capability to actively choose to participate in care in a way that is consistent with the individual’s values and preferences in cooperation with a health care provider or institution for the purposes of improving clinical outcomes or experiences with care [1,25]. When applied to mHealth apps, the literature has defined patient engagement to have an objective component assessing the amount, frequency, duration, and depth of usage in addition to a subjective component characterizing the user’s overall experience with the technology [9]. Based on these definitions, our team created a data extraction tool utilizing the Mobile Application Rating Scale (MARS), which was developed by the Queensland University of Technology [24-26]. We selected MARS to assess patient engagement because it evaluates the quality of mHealth app’s useability based on 22 items within 5 information technology parameters of (1) engagement, (2) functionality, (3) aesthetics, (4) information, and (5) subjective quality [24]. These 5 parameters align with the objective and subjective components of how patient engagement is defined within the mHealth technology literature [9,24]. MARS has a specific patient engagement parameter assessed through 5 items: (1) entertainment, (2) interest, (3) customizability, (4) interactivity, and (5) relevance to its target group. When creating the patient engagement section of the data extraction tool, our team determined all 5 MARS parameters were necessary to capture both objective and subjective components of patient engagement as applied to mHealth apps [9]. Following the patient engagement parameter within the MARS is functionality, which is evaluated through 3 items: (1) technical performance, (2) ease of use, navigation, and (3) general design [24]. Aesthetics has 3 items that assess the app’s (1) layout, (2) graphics, and (3) visual appeal [24]. Information is assessed through 6 items that include examining the accuracy, quality, and quantity of credible knowledge in the app [24]. Subjective quality has 4 items assessing whether users would recommend the app to other people, and the users’ overall rating of the app [23]. Each parameter of the 5 parameters within MARS is rated on a scale of 1-5 (1: inadequate, 2: poor, 3: acceptable, 4: good, and 5: excellent). The data extraction tool contained all 5 parameters with the rating scale included [24].

Patient activation is the patient’s willingness and ability to take behavioral actions to manage their health [9]. When assessing mHealth apps, patients must perceive that the app has the features they desire to support them in taking behavioral actions to collaborate with providers and manage their RA between clinical visits [9]. To date, there are measures for evaluating patient activation resulting from interventions (ie, Patient Activation Measure and Patient Health Engagement Scale.) Yet, to our knowledge, no methodology exists to apply a priori to evaluate app functionality to promote such activation prior to app adoption. Thus, when creating our data extraction process for activation, we applied social cognitive theory (SCT), which describes how individuals internalize their experiences along with the actions of others and influences from the environment to adopt new health behaviors [27,28]. When applied to RA, the theory specifies that a person’s knowledge of their disease and self-efficacy beliefs operate together with goals for living to form expectations about treatment that, in turn, foster collaboration with providers and treatment adherence. During our data extraction process, our team developed the patient activation portion of the data extraction based on SCT [27,28]. Patients with RA were included on the research team because they provide the patient perspective in study design, data analysis, and interpretation of the findings [9,29-31]. Our team worked iteratively with patients, meeting biweekly to gain feedback to develop the patient activation portion of the data extraction tool.

The patient activation portion of the data extraction tool contained the 6 categories of SCT [27,28]: (1) knowledge, (2) perceived self-efficacy, (3) outcome expectations, (4) goal formation, (5) sociostructural factors, and (6) self-regulation [24]. These categories have constructs within them that are directly related to important components necessary to foster behavioral change [27,28]. Knowledge contains 1 construct: inclusion of educational resources to provide information about the disease and treatment. Perceived self-efficacy contains 4 constructs that examine the translation of personal experiences and social persuasion into beliefs about treatment and disease control. Outcome expectations has 3 constructs related to assisting patients form expectations about their disease control. Goal formation has 2 constructs related to helping patients identify goals relevant to treatment decisions. Sociostructural factors has 2 constructs related to social and environmental factors that exist outside of the individual’s control. Self-regulation has 4 constructs related to medication adherence and following through on other relevant behaviors for disease management. The data extraction tool for patient activation contained the 6 categories of SCT and the subconstructs with their definitions.

### Data Analysis

From December 2020 to May 2021, 2 independent raters (authors MC and HT) evaluated the final set of app features for patient engagement and activation using content analysis and applying the data extraction tool. They completed the patient engagement portion of the data extraction tool by scoring each of the 5 MARS parameters according to the scale described in the Data Extraction section. When comparing the independent ratings of the parameters, they noted only 5 differences among the parameter ratings. These were discussed, and a total MARS score was calculated following the literature [24]. Our team met to collectively discuss the results from the reviewers' assessments.

The 2 reviewers also independently applied the patient activation portion of the data extraction tool to the final set of apps. The tool allowed them to conduct a content analysis of each feature determining whether the definitions of SCT categories and constructs were present [32-36]. After the independent analysis, the reviewers met to discuss any discrepancies and achieve consensus about which features related to which construct/category to ensure high interrater reliability [31]. Consensus was achieved through iterative discussions about what a particular construct/category meant and how a feature within an app represented it. After achieving consensus among the reviewers, the rest of our research team met with the reviewers and 2 patients with RA for a further discussion about features to finalize the determination of how features aligned with constructs/categories [32-35]. Constructs coded as “present,” based on app features, were summed to determine the total number of SCT constructs within each of the 6 categories for each app [27,28].

To determine the quality of the app for patient activation, our team used the results from the content analysis to develop an SCT ratio. The ratio was calculated by dividing the number of constructs identified within the app by the total number of app features. An app with a social cognitive ratio of 1 meant that it displayed an equal number of constructs as compared to app features, which suggests a good app for patient activation because approximately every feature within the app relates to a construct. Apps with an SCT ratio higher than 1 represented a high-quality app for patient activation because many features within the app relate to more than 1 theoretical construct. This ratio was important to patients with RA who worked with our team. They felt the ratio helped identify good and high-quality apps that contained features facilitating patient activation aligned with SCT. Further, the ratio helped identify apps that were more streamlined and did not contain other functionalities that distracted from focus on the adoption of health behaviors supporting PCC. Apps were ranked based on the calculated SCT ratio.

The results of the engagement and activation analyses were plotted on a perceptual map to determine each app’s ability to facilitate PCC. Perceptual mapping is a useful technique to evaluate how products compare relative to consumer perceptions and product attributes [36]. The perceptual map plots engagement on the horizontal axis using the MARS score and activation on the vertical axis using the SCT ratio. Apps with a higher MARS score and higher SCT-to-total feature ratio demonstrate a greater ability to facilitate PCC in the clinical management of RA.

## Results

### Identification

**Figure 1:**
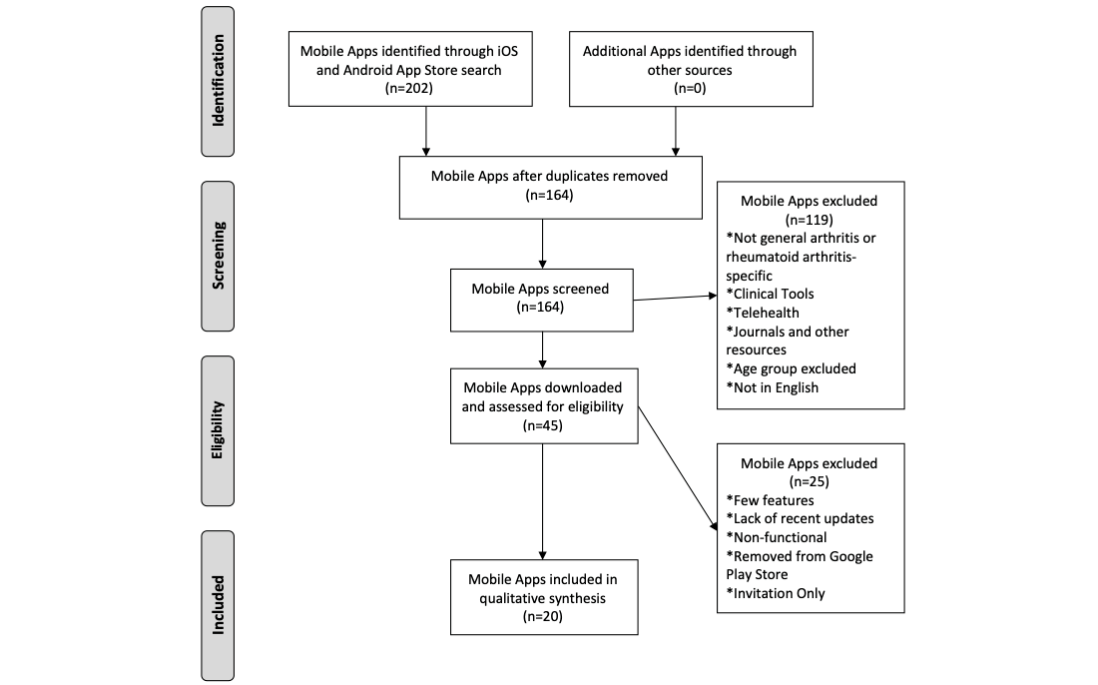
Identification process of mobile applications for rheumatoid arthritis in the United States.

**Figure 2:**
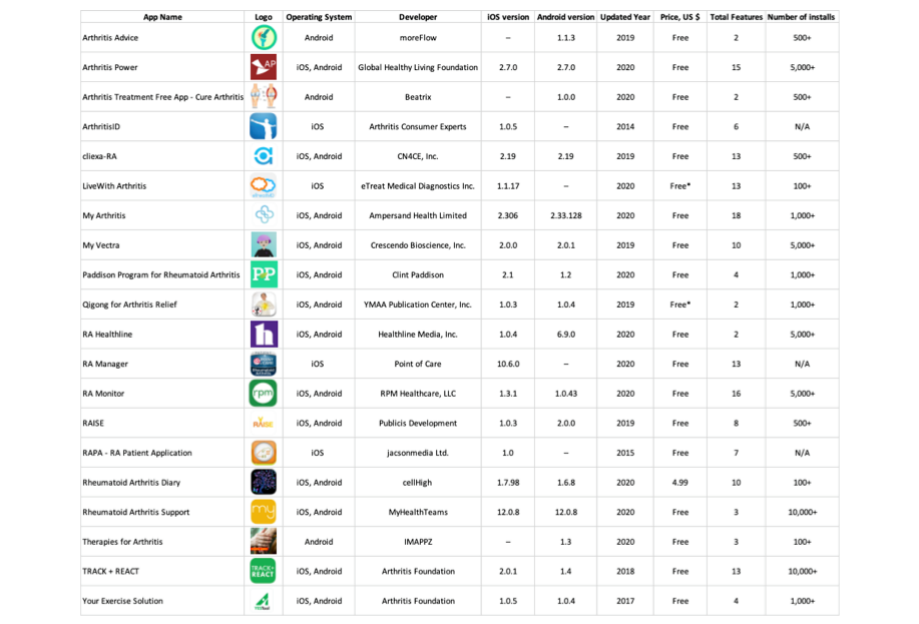
Operating characteristics of US rheumatoid arthritis mobile apps. N/A: not applicable; RA: rheumatoid arthritis.

The initial search of key words yielded an original sample of 202 mobile apps from Google Play and Apple App stores (Figure 1). After 38 duplicate apps were removed, 164 remained. Of those, 119 apps met the exclusion criteria and were removed from the sample prior to downloading from the Google Play or Apple App stores. The 45 remaining apps were downloaded and assessed for further eligibility in the study. Of the downloaded apps, 25 met further exclusion criteria, and 20 remained for analysis. The operating characteristics of the remaining apps show that all apps were developed (Figure 2). Among the apps, 12 were available on both Android and iOS operating systems. In addition, 16 were updated in the last 3 years, and 19 were freely available to patients with no monetary cost for the app needed upon download (Figure 2).

### Patient Engagement

**Figure 3:**
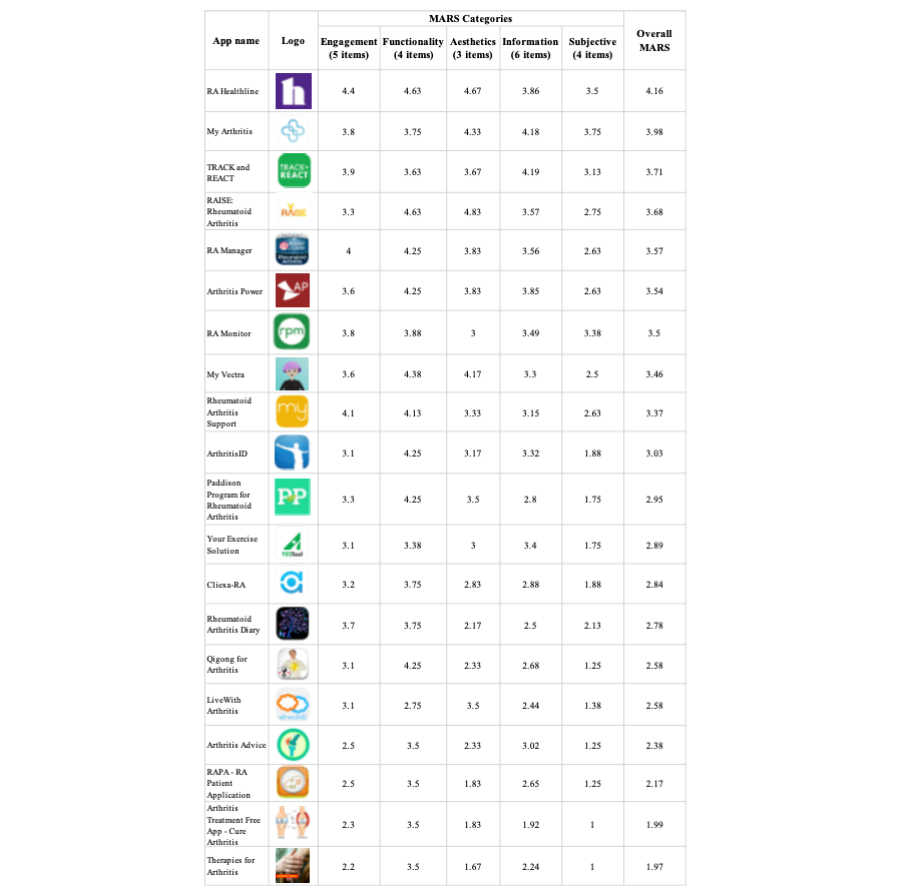
Patient engagement evaluations of rheumatoid arthritis mobile apps using the Mobile Application Rating Scale (MARS). Apps appear based on their overall MARS score from highest to lowest. MARS: Mobile Application Rating Scale; RA: rheumatoid arthritis.

For the patient engagement analysis, each app’s score for the 5 parameters (engagement, functionality, aesthetics, information, and subjective), along with the overall MARS score is shown (Figure 3). The percentage of apps rated as good (ie, score greater than 4 and less than 5) varied with the parameter, with 15% (n=3) so rated for engagement, 45% (n=9) for functionality, 20% (n=4) for aesthetics, and 10% (n=2) for containing information helpful to patients. In terms of the subjectivity parameter, only 10% (n=2) had scores indicating acceptability (ie, score greater than 3 and less than 4), with the user indicating they would recommend the app to other people. For the overall MARS score, only 1 app scored greater than a 4, indicating at least a good rating across all 5 parameters.

### Patient Activation

**Figure 4:**
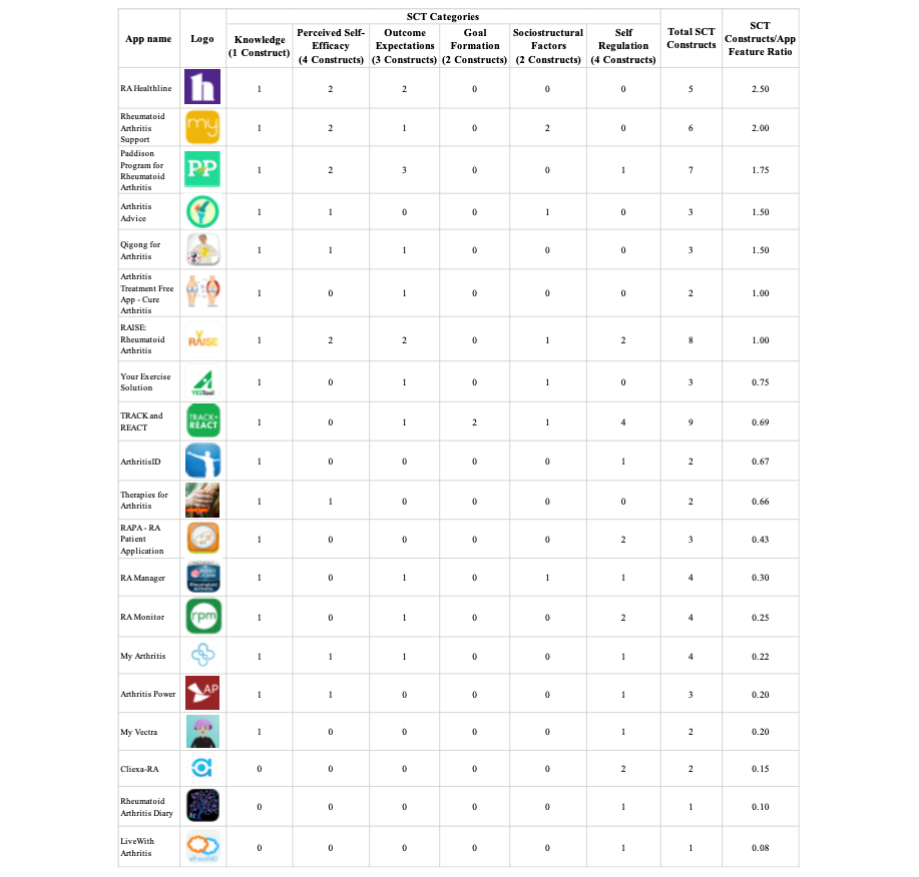
Patient activation evaluation of rheumatoid arthritis mobile apps using social cognitive theory. RA: rheumatoid arthritis; SCT: social cognitive theory.

The results of the patient activation analysis based on SCT are displayed (Figure 4). Among the apps, 85% (n=17) improved patient knowledge of the disease through the inclusion of educational resources, and 45% (n=9) promoted self-efficacy toward treatment by having at least 1 of 4 constructs focusing on the translation of experiences and social persuasion into beliefs about disease control. Over half of the apps (n=11, 55%) included features that helped patients form expectations about their disease control. Only 1 app (5%) included a goal formation feature. Moreover, 30% (n=6) of the apps addressed sociostructural factors that exist outside of the individual’s control. Slightly over half (n=12, 60%) of the apps included features for improving self-regulation through monitoring of the disease or symptoms. While many apps contained a few features that align with SCT, only 25% (n=5) contained 5 or more of the 16 constructs, and no app had features within all 6 categories. In terms of the quality of the app for patient activation, 2 apps (10%) had a social cognitive ratio equal to 1, meaning the app displayed an equal number of constructs as compared to total app functions. This suggests a good app for patient activation because each function within the app relates to a construct. Five (25%) apps had an SCT ratio higher than 1, representing high-quality apps for patient activation because the app had features within it relating to more than one 1 construct. Of those 5 apps, 2 (10%) had a ratio of 2 or higher, meaning many features within the app represented multiple SCT constructs (Figure 4).

### Patient-Centered Care

**Figure 5:**
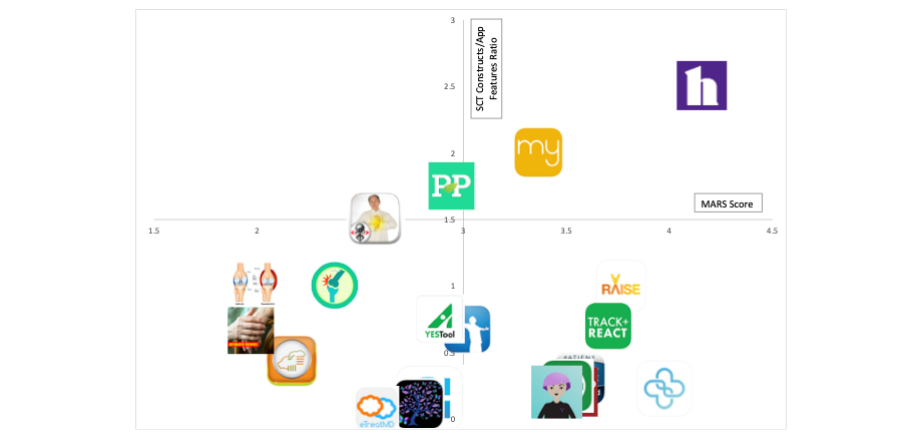
Perceptual map of US mobile apps' ability to facilitate patient-centered care. MARS: Mobile Application Rating Scale; SCT: social cognitive theory.

The perceptual map demonstrates each app’s ability to facilitate PCC through patient engagement (ie, MARS score) and patient activation measured by the SCT feature to overall feature ratio (Figure 5). Apps in the upper right quadrant demonstrate the greatest patient-centeredness per these 2 dimensions. RA Healthline had both a good ability to foster patient engagement (MARS score 4.16) and the highest patient activation assessment (SCT feature to total feature ratio of 2.5). Additionally, Rheumatoid Arthritis Support was located in the upper right quadrant. It had a MARS score of 3.17, indicating acceptable patient engagement and an SCT to overall app feature ratio of 2. These 2 apps stood out among the rest because they were able to score highly in terms of useability needed for patient engagement via the MARS. They also had features that satisfied multiple categories and SCT constructs. Patients on our team noted that this also allowed them to be efficient in their design with respect to patient activation since they had higher SCT-to-total feature ratios. As shown in Figure 1, a number of other apps scored highly in patient engagement (ie, MARS) but scored lower in terms of patient activation, as measured by the number of features demonstrating SCT content to overall feature ratio (lower right quadrant).

## Discussion

### Principal Findings

To our knowledge, this is the first evaluation of mHealth apps for RA to assess their ability to facilitate PCC. In PCC, there is a role for both the patient and the provider with shared decision-making at the point of care. As an initial step, such apps must foster patient engagement and activation to enhance shared decision-making. Our findings demonstrate there are few mobile apps available within the United States that contain the necessary features to adequately support patients as active partners in their care. Specifically, only 2 apps emerged as having an acceptable or good ability to foster patient engagement and having quality content to promote patient activation, which are 2 necessary components to supporting the patient’s role in PCC. However, both these apps lacked a goal-setting feature that is important to integrate patient values into clinical decisions that guide PCC.

### Comparison With Prior Work

These findings are consistent with previous reviews of RA apps that are largely focused on useability and self-management. Those reports also found the quality and content of the available mHealth apps to be highly variable. For example, studies found a lack of high-quality mobile apps that provide a comprehensive user experience or longitudinal disease tracking that aligned with clinical guidelines [10,13]. Additionally, few use validated questionnaires or even have the ability to support important aspects of clinical management such as physical activity [11,12]. Moreover, even with the more limited focus on self-management, the efficacy of the available apps is largely uncertain [7]. Overall, the general findings of these reviews are that most apps are of low-to-moderate quality and need more emphasis on working with patients and providers in their development [37].

Our study extends this previous work by evaluating features in their ability to engage the patient and support their activation to facilitate PCC of RA. Specifically, our assessment was guided by SCT, which contains constructs that enable patient activation [27,28]. Both engagement and activation are necessary for patients to effectively collaborate with their rheumatologist to reach shared therapeutic decisions. As patients continue to adopt mHealth apps, we recommend (as others have) that patients, as the end users of the app, be involved in the selection of desired functions and the app design to ensure both dimensions of patient engagement and activation are adequately met [10-13,37]. Additionally, if PCC for RA is to be achieved, app functionality in the areas of goal formation and preferences for symptom and side effect management is critical. Goal formation and identification of treatment preferences are central to how patients approach the treatment selection process. Features that support patients in these areas, along with disease tracking and recording of problematic symptoms and side effects, may enable more efficient and effective discussions surrounding treatment selection, leading to improved outcomes. A pragmatic approach to development is needed to balance the necessary features needed for patient engagement and activation against development costs. Development cost considerations are important to ensure that mobile apps for RA remain free for patients to use. App features need to be created that can promote multiple parameters of patient engagement via the MARS and multiple constructs of SCT to facilitate efficient app use. Future research should focus on establishing the efficacy of mobile apps for RA in terms of the sustainability of use that is necessary to provide clinically relevant information. Additionally, focus should be placed on the activation mechanism to determine if and how apps impact decision-making, outcomes, and the clinical workflow to ensure its translation into clinical practice.

### Strengths and Limitations

There were several strengths of this review. First, we analyzed each mobile app’s ability to promote PCC through the necessary components of patient engagement and activation. Following other studies, we used the MARS to assess patient engagement. SCT from the health promotion and education literature was used to evaluate the quality of the content of the apps for patient activation. A novel decision extraction tool was developed by which to evaluate the extent to which mHealth apps for RA utilize SCT. Additionally, our team relied on patients who had RA to design the study, code, review, and interpret the findings. One limitation of our study is that the review only encompassed mobile apps available in US mobile app stores. Further, this review focused only on mobile apps for RA and arthritis, but patients may use apps designed for other diseases, pain, or alternative medical approaches to managing this disease. This review also focuses on reviewing app contents for the ability to potentially foster patient engagement and activation. There are also limitations in the MARS, in that it focuses on app quality and useability; however, it is applicable to evaluating patient engagement of mHealth technology [9,24]. As noted, future research is needed to evaluate the efficacy of apps in terms of patient engagement and activation as outcomes of an intervention using these apps.

### Conclusions

Patient-centered care of RA aligns patients’ goals for living with their preferences for symptom and side effect management to enable the selection of a therapy that promotes greater adherence and more effective disease control. We found that there are only 2 mobile apps available within the United States that rate as acceptable or good in terms of patient engagement and activation, which are 2 dimensions necessary for facilitating PCC. As the prevalence of mobile apps expands, the design of these mobile apps needs to include patients to ensure their engagement and activation. Physicians also are critical to ensure that clinically relevant information is being collected and used in decision-making. Areas for further investigation of mHealth apps include their impacts on patient engagement, activation, treatment decisions, and disease trajectory.

## Abbreviations

MARS: Mobile Application Rating Scale
mHealth: mobile Health
PCC: patient-centered care
PRISMA: Preferred Reporting Items for Systematic Reviews and Meta-analyses
RA: rheumatoid arthritis
SCT: social cognitive theory
